# Assessment of Agreement between Two Difference Prostate-Specific Antigen Assay Modalities

**DOI:** 10.3390/biology10040297

**Published:** 2021-04-05

**Authors:** Jae Hoon Chung, Minsu Park, Hyun Cho, Wan Song, Minyong Kang, Hyun Hwan Sung, Hwang Gyun Jeon, Byong Chang Jeong, Seong IL Seo, Hyun Moo Lee, Seong Soo Jeon

**Affiliations:** 1 Samsung Medical Center, Department of Urology, Sungkyunkwan University School of Medicine, Seoul 06351, Korea; dr.jhchung@gmail.com (J.H.C.); waniyo25@hanmail.net (W.S.); m79.kang@samsung.com (M.K.); hhsunguro@gmail.com (H.H.S.); hwanggyun.jeon@samsung.com (H.G.J.); bc2.jung@samsung.com (B.C.J.); siseo@skku.edu (S.I.S.); hyunmoo.lee@samsung.com (H.M.L.); 2 Department of Statistics, Keimyung University, Daegu 42403, Korea; minsu.park51@gmail.com; 3 Statistics and Data Center, Samsung Biomedical Research Institute, Samsung Medical Center, Seoul 06351, Korea; hyun09.cho@sbri.co.kr

**Keywords:** prostate-specific antigen, diagnosis, statistics

## Abstract

**Simple Summary:**

Prostate-specific antigen is a biomarker for prostate cancer. If the level of prostate-specific antigen is high, a prostate biopsy is needed to diagnose prostate cancer. However, the definite level of prostate-specific antigen that requires prostate biopsy has not been established. Currently, there are many kinds of assay modalities that have been used for prostate-specific antigen testing. This study was conducted under the hypothesis that there will be differences between different assay modalities; therefore, there is no definite prostate-specific antigen level for prostate biopsy. In our study, the level of prostate-specific antigens was measured in one blood sample per patient, with two different assay modalities in 4810 patients. As a result, we confirmed that the overall agreement between the two modalities is excellent, but the agreement is slightly different in some ranges that may give clinical significance. Accordingly, the conformity between each assay modality should be secured in the future, and the threshold for the level of prostate-specific antigens for biopsy by each assay modality should be independently determined.

**Abstract:**

There is controversy over the usefulness of prostate-specific antigen (PSA) as a prostate cancer (PCa) biomarker. This controversy arises when there are differences in the results of PSA assay modalities. In this study, which aimed to evaluate a proper validation between the two PSA assay modalities, the agreement between the results of the two modalities was analyzed. PSA examinations were conducted using two PSA assay modalities in 4810 patients. The intra-class correlation coefficient (ICC) and weighted kappa analysis were used to evaluate the agreement between the two assay modalities. A linear regression was performed to evaluate the association between the two assay modalities. According to ICC values (ICC: 0.999, *p* < 0.001) and weighted kappa analysis values (kappa: 0.951, alpha’s standard error (ASE): 0.001, *p* < 0.0001), the agreement between the assay modalities was rated as excellent. However, the strength of agreement was poor in the following PSA sub-groups: 0.05–0.1 ng/mL (ICC: 0.281, *p* = 0.0860); 0.15–0.2 ng/mL (ICC: 0.288, *p* = 0.0036); 1.5–2.0 ng/mL (ICC: 0.360, *p* = 0.0860); and 2.0–2.5 ng/mL (ICC: 0.303, *p* = 0.0868). In linear regression analysis, when modality B PSA yielded a value of 0.2 ng/mL, the expected value for modality A was 0.258 ng/mL (95% CI: 0.255–0.260), and when modality B PSA yielded a value of 4 ng/mL, the expected value for modality A was 3.192 ng/mL (95% CI: 3.150–3.235). The difference in the PSA values between the two PSA assay modalities is confirmed, and this difference may be clinically meaningful.

## 1. Introduction

Prostate-specific antigen (PSA) was first introduced by Wang et al. in 1979 [1]. PSA is a glycoprotein produced by the epithelial component of the prostate gland. Various prostatic diseases, such as prostate cancer (PCa), benign prostatic hyperplasia, and acute prostatitis, cause structural distortion of the prostate gland, which lead to the enhancement of the production of PSA. Stamey et al. reported a correlation between PCa volume and PSA in 1987 [2]. From this point on, PSA began to be recognized as the most important serum biomarker associated with PCa. In addition, in 1991, Catalona et al. reported that PSA was superior to PCa detection compared to digital rectal examination and proposed it as a useful tool for screening PCa [3]. Prostatic biopsies are considered a gold standard for diagnosis of PCa. However, prostatic biopsies are generally performed in patients who have increased levels of PSA. Traditionally, if the PSA value exceeds 4 ng/mL, there is a possibility of PCa, and additional tests such as transrectal prostate biopsy or magnetic resonance image are recommended [4]. However, the PSA cut-off value of 4 ng/mL is still controversial. Many studies have suggested that the PSA cut-off value for PCa set as 4 ng/mL is too high. In 1994, Littrup et al. reported that the PSA cut-off value should be lowered to the level of 3 ng/mL [5], and several studies have since suggested that the PSA cut-off value can be reduced to 2.5 ng/mL [6,7]. In particular, Kim et al. reported that there was no significant difference in the detection rate of PCa when they compared the group of PSA levels with 2.5–4.0 ng/mL and those with 4.0–10.0 ng/mL [8]. In summary, there is still no definite PSA cut-off value for prostate biopsy for the detection of PCa. Moreover, PSA is not only used in PCa screening but also in the monitoring of disease progression in an untreated group and in the evaluation of treatment response [2,9,10,11,12]. After the treatment of PCa, using radical prostatectomy, radiation therapy, or high-intensity focused ultrasound, the PSA value is measured to confirm the treatment outcomes. The PSA value may also help to diagnose recurred cancer by increasing its level. Biochemical recurrence (BCR) after radical prostatectomy is diagnosed when the PSA level is measured to be 0.2 ng/mL or higher. Therefore, PSA in post-treatment follow-up has clinical significance at a very low level, and the accuracy and reliability of the PSA test are required.

When the PSA assay was first developed, it could be reported only in the range of 0.3–0.6 ng/mL, which was not clinically useful [12,13]. However, since then, many advances in technology have made it possible to confirm PSA levels even below 0.2 ng/mL, and recently, equipment that can report even 0.001 ng/mL has been developed [14]. In spite of technological development and the many PSA assay modalities that have been introduced, there is still controversy over the PSA cut-off value for PCa and the usefulness of PSA as a PCa biomarker. Although there may be several factors that contribute to this controversy, a premise is required to clearly define the clinical significance of PSA level. The premise is that the results of a PSA assay must be equal between all assay modalities. However, there are differences among PSA assay modalities at each medical institution, and there has been no report on the consistency between PSA assay results by different PSA assay modalities. The most important information on whether each PSA assay modality measurement has been properly validated has not yet been obtained. Therefore, in this study, PSA values were measured using two independent PSA assay modalities from a single sample per patient, and correlation evaluation was performed to confirm the agreement between the two modalities.

## 2. Materials and Methods

### 2.1. Study Design and Patients

From October 2019 to December 2019, PSA examinations were conducted using two PSA assay modalities drawn from one sample for all patients requiring PSA examinations at the Urology department. In our institution, before the change of PSA assay modality, PSA measurement was performed using two assay modalities in the same patients to evaluate the reliability of the new assay modality and reduce the possible confusion in the clinic. There was no additional cost for the patients. A total of 5302 tests were conducted, and in order to maintain statistical independence, the trials repeated by the same patient were excluded. Therefore, the results of the PSA values of 4810 patients were analyzed.

PSA measurement was performed within 8 h of sampling, routinely. In most cases, it was stored at room temperature (20–25 °C). When test could not be performed within 8 h, blood samples were stored in a refrigerator of 2–8 °C. Frozen samples were not used.

### 2.2. PSA Assay Modalities

The PSA assays used in this study were ADVIA Centaur^®^ XP, Siemens Healthcare Diagnostics Inc., Deerfield, IL, USA (modality A) and Cobas e 801, Roche Diagnostics GmbH, Mannheim, Germany (modality B). The measuring range of PSA assay modality A is 0.01 to 100 ng/mL and that of modality B is 0.006 to 100 ng/mL. Calibration of the assay was performed according to the recommendation of both companies (Siemens Healthcare Diagnostics Inc and Roche Diagnostics GmbH).

### 2.3. Statistical Analysis

The intra-class correlation coefficient (ICC) was used to evaluate the agreement between the two assay modalities. After that, through sub-grouping (G1: 0.01–0.05, G2: 0.05–0.1, G3: 0.1–0.15, G4: 0.15–0.2, G5: 0.2–0.5, G6: 0.5–1.0, G7: 1.0–1.5, G8: 1.5–2.0, G9: 2.0–2.5, G10: 2.5–4.0, G11: 4.0~), the agreement between each PSA numerical group was also assessed using the ICC procedure. In addition, categorization was conducted through sub-grouping, and the degree of agreement was further evaluated using weighted kappa analysis. A linear regression was performed to evaluate the association between the two assay modalities.

Statistical analyses were performed using SAS version 9.4 (SAS Institute, Cary, NC, USA) and R 3.6.1 (Vienna, Austria; http://www.R-project.org/, accessed on 11 February 2020).

### 2.4. Ethics Statement

The study was performed in agreement with applicable laws and regulations, good clinical practices, and ethical principles as described in the Declaration of Helsinki. The Institutional Review Board of Samsung Medical Center approved the present study (approval no. 2020-01-155-001). Informed consent was waived by the Board.

## 3. Results

In a total of 4810 patients, the mean age was 67.73 ± 9.35 years (age range: 21 to 97 years). Among them, 2431 (50.54%) were PCa patients. PSA measurements were performed on both assay modalities simultaneously from a single sample per patient. In order to evaluate the agreement between the two assay modalities, ICC values were obtained between modalities using the entire dataset for each, and the strength of agreement was excellent (ICC: 0.999, *p* < 0.001). In addition, ICC values between modalities were also obtained by PSA sub-group based on modality A. The strength of agreement between the PSA value of the 0.2–0.5 ng/mL group (ICC: 0.862, *p* < 0.001) and that of the over 4.0 ng/mL group (ICC: 0.999, *p* < 0.001) was excellent. However, the strength of agreement was poor in the following PSA sub-groups: 0.05–0.1 ng/mL (ICC: 0.281, *p* = 0.0860); 0.15–0.2 ng/mL (ICC: 0.288, *p* = 0.0036); 1.5–2.0 ng/mL (ICC: 0.360, *p* = 0.0860); and 2.0–2.5 ng/mL (ICC: 0.303, *p* = 0.0868) (Table 1).

Weighted kappa analysis values were 0.951 (95% confidence interval (CI): 0.948–0.954, alpha’s standard error (ASE): 0.001, *p* < 0.0001), and the agreement between the assay modalities was rated as excellent (Table 2).

**Figure 1:**
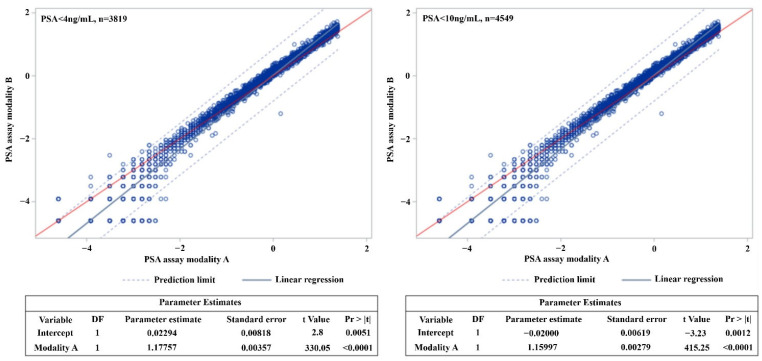
Logistic regression according to two prostate-specific antigen (PSA) assay modalities. The point where the regression line meets the straight line with a slope of 1 in PSA < 4 ng/mL group: 0.878808304. The point where the regression line meets the straight line with a slope of 1 in PSA < 10 ng/mL group: 1.133175017. DF: degrees of freedom, Pr: probability.

Linear regression analysis after excluding an outlier case (where the PSA value was over 10 ng/mL) for modality A showed that the regression line met the axis at 1.133 (slope of 1), and the parameter estimate was 1.160 (standard error (SE): 0.003). Furthermore, based on modality A, except for an outlier case (where the PSA value exceeded 4 ng/mL), the point where the regression line met the axis was 0.879 (slope of 1), and the parameter estimate was 1.178 (SE 0.004) (Figure 1). In addition, the values predicted for modality A compared to modality B are shown in Table 3. If modality B PSA yielded a value of 0.2 ng/mL, the expected value for modality A was 0.258 ng/mL (95% CI: 0.255–0.260); the expected value at 3 ng/mL (modality B) was 2.507 ng/mL (modality A, 95% CI: 2.476–2.539); and at 4 ng/mL (modality B), the expected value was 3.192 ng/mL (modality A, 95% CI: 3.150–3.235) (Table 3).

## 4. Discussion

This study showed that the overall correlation between the PSA values of the two assay modalities was consistent between assays. However, as a result of sub-analyses to identify clinical implications, it was confirmed that there were poor agreements at specific points of PSA value.

The PSA value is widely used as a parameter for PCa detection and assessment. Traditionally, the PSA cut-off value for PCa detection was set to 4 ng/mL, but in this case, the specificity has been shown to be 21%, and sensitivity is 91% [15]. This can lead to high false positive rates and unnecessary prostate biopsies, and finally, to an increase in the frequency of complications due to unnecessary prostate biopsies [16]. Because of these limitations of PSA, many other additional tests have been suggested, e.g., free PSA, prostate health index, 4Kscore, PCA3, Select MDx, and ExoDx Prostate [17,18,19,20,21,22]. In addition, recently, prostate magnetic resonance imaging and prostate-specific membrane antigen positron emission tomography have also been used [23,24]. However, performing these additional tests is also determined based on the PSA cut-off value of 4 ng/mL, and they require additional economic burden for both patients and society. Therefore, a definite PSA cut-off value is important not only because it determines the prostate biopsy at risk of complications, but also because it may have an influence on the occurrence of medical opportunity cost.

However, several studies have reportedly lowered the PSA cut-off value to 2.5 or 3 ng/mL [5,6,7]. The basis for these reports was that the prevalence of PCa was not low, even when the PSA value was below 4 ng/mL. Although PSA has been used as a parameter of Pca for over 30 years, there is still a controversy over the cut-off value for Pca detection, and this controversy raises a concern with the effectiveness of PSA itself.

As a result of the present study, when the PSA value of modality B was 4.0 ng/mL, the estimated value in modality A was 3.19 ng/mL. In addition, when the PSA value obtained with modality B was 3.0 ng/mL, the estimated value in modality A was 2.51 ng/mL. By assessing these results, it is possible to predict whether the claims for the PSA cut-off value for Pca are different among researchers because the used PSA assay modality was different among researchers. In the present study, although the overall agreement between the two assay modalities was excellent, clinical decision-making can be affected, even when the PSA value changes by as little as 0.1 ng/mL. Therefore, it can be suggested that statistical validation and clinical validation produce different results. Although this study was not able to evaluate the sensitivity and specificity of the two modalities for PCa detection, we could suggest the reason why there had been no definite PSA cut-off value for PCa screening.

In our institution (Samsung Medical Center, Seoul, Korea), we have used modality A to measure PSA for a long time. The studies, which were conducted in our institution using modality A, showed that the PCa detection rate was 21.8 to 25.0% in patients with PSA of 2.5 to 4 ng/mL [8,25]. Moreover, among them, over 75% of the patients were diagnosed with clinically significant PCa [25]. Based on these data, we set a PSA value of 2.5 ng/mL as the cut-off value for PCa screening. The present study was conducted when the PSA assay modality was converted to modality B, and it is expected that the PSA cut-off value will be adjusted to 3 ng/mL through this analysis. In addition, when the PSA value in modality B is 3 to 5 ng/mL, a similar PCa detection rate of 2.5 to 4 ng/mL in modality A is expected.

This difference between PSA assay modalities can also affect the determination of biochemical recurrence after radical prostatectomy and evaluation of recurrence after radiation therapy or high-intensity focus ultrasound. The American Urological Association and the European Association of Urology panel have defined the BCR as above 0.2 ng/mL [26]. Several previous studies tried to reduce cancer-specific mortality by predicting BCR and performing rapid adjuvant/salvage treatment after prostatectomy [27,28,29]. However, the results of the present study showed that when the PSA value of modality A was increased by 1, the result seen in modality B was confirmed to increase by 1.160 (in the PSA < 10 ng/mL group) or 1.178 (in the PSA < 4 ng/mL group). In addition, when the PSA value was 0.2 ng/mL for modality B, the estimated value for modality A was 0.25 ng/mL. Although it is impossible to definitively determine which assay modality is best for cancer evaluation, assuming that a relatively high value is a true result, there is a problem because the diagnosis of recurrence might be underestimated in the group tested with a modality that yields relatively low values. Obviously, it is important in the clinic to assess sensitive changes in PSA over time using repeat PSA measurements, PSA doubling time, and PSA velocity. However, if the absolute value is different for each assay modality, this will be an important factor to be sufficiently controlled by clinicians.

In this study, among the enrolled patients, 50.54% (2431/4810) were diagnosed with PCa. In PCa patients, 57.14% (1389/2431) had a PSA value of lower than 0.1 ng/mL after receiving definite treatment such as radical prostatectomy. A low ICC was confirmed in a specific section with a low PSA value. The lower limit of the measurement range was 0.006 ng/mL for modality A and 0.01 ng/mL for modality B, and both assay modalities were calibrated according to the schedule and method specified by each company. Therefore, low ICC might be caused by a difference between the values measured by the two assay modalities rather than the measurement error at low values. Low ICC is a significant finding, because a small difference in PSA at low levels can affect the patient’s treatment strategy and prognosis during follow up.

The problem presented in this study will not cause clinical problems in real practice if the PSA cut-off value is independently determined for each clinical center and treatment is performed based on long-term medical records and statistical analysis maintained by each center. However, confusion may arise when a patient is transferred to another institution that uses a different PSA assay modality. Moreover, it is difficult to propose a globally agreed upon PSA modality guideline. Furthermore, medical information, which is difficult to agree on, can disrupt public health practices at the present time, because it is common for conflicting information to be spread through the Internet. In addition, confusion may occur in treatment decisions when the PSA assay modality is changed, even in a single clinical center. Recently, a controversy has been reported with respect to the usefulness of PSA as a biomarker [30]; this may be due to the lack of meticulous validation between PSA assay modalities.

A key limitation of this study is that it is retrospective. However, in order to prevent other biases, only the PSA values measured by two assay modalities drawn from a single sample for each person were assessed. As this was not a comparative study, it was not possible to evaluate superiority and efficiency between the two assay modalities. In addition, as the range of the measured value became wider, an error may have occurred in the regression analysis, so correlation was obtained for PSA values when they were less than 4 ng/mL and less than 10 ng/mL. In addition, the overall comparison between the two modalities was rated excellent in terms of concordance. Because this study did not assess the clinical implications, it was impossible to determine which of the two modalities yielded “true” results. Well-designed, large-scale, prospective studies will be required for assessing efficiency, sensitivity, and specificity of PCa detection according to PSA assay modalities. However, this study suggests, for the first time, that there may be differences between PSA assay modalities and cut-off points in terms of clinical significance. Thus, these results indicate the current medical situation in terms of treatment and biopsy decisions, and they may be clinically helpful.

## 5. Conclusions

Through this study, the overall agreement between two PSA assay modalities was confirmed. However, it was also confirmed that there was a difference in the result values of the two PSA assay modalities, and that the difference may be clinically meaningful. This result may have been the cause of controversy over the clinical usefulness of the PSA values, and, therefore, the cut-off value of PCa may not be established.

## Figures and Tables

**Table 1:** Analysis of agreement between two PSA assay modalities.

PSA Modality A	N	ICC	95% CI		*p* Value
			Lower	Upper	
0.01~0.05	1012	0.092	−0.045	0.228	0.1063
0.05~0.1	377	0.281	−0.085	0.558	0.0860
0.1~0.15	119	0.492	0.333	0.622	<0.0001
0.15~0.2	86	0.288	0.081	0.471	0.0036
0.2~0.5	382	0.862	0.705	0.923	<0.0001
0.5~1.0	531	0.774	0.130	0.913	0.0103
1.0~1.5	315	0.488	−0.064	0.754	0.0478
1.5~2.0	249	0.360	−0.095	0.660	0.0860
2.0~2.5	184	0.303	−0.090	0.592	0.0868
2.5~4.0	564	0.594	−0.085	0.845	0.0620
4.0~	991	0.999	0.999	0.999	<0.0001
Total	4810	0.999	0.999	0.999	<0.0001

Intra-class correlation coefficient.

**Table 2:** Analysis of the agreement between two PSA assay modalities.

	Modality B	G1	G2	G3	G4	G5	G6	G7	G8	G9	G10	G11	Sum
Modality A													
G1		1008 (99.6)	4 (0.4)										1012
G2		241 (63.93)	126 (33.42)	10 (2.65)									377
G3		2 (1.68)	34 (28.57)	71 (59.66)	12 (10.08)								119
G4			3 (3.49)	16 (18.6)	51 (59.3)	16 (18.6)							86
G5					12 (3.14)	314 (82.2)	56 (14.66)						382
G6						6 (1.13)	402 (75.71)	123 (23.16)					531
G7						1 (0.32)	1 (0.32)	231 (73.33)	80 (25.4)	2 (0.63)			315
G8								4 (1.61)	133 (53.41)	109 (43.78)	3 (1.2)		249
G9									2 (1.09)	90 (48.91)	92 (50)		184
G10											378 (67.02)	186 (32.98)	564
G11											1 (0.1)	990 (99.9)	991
Sum		1251	167	97	75	337	459		215	201	474	1176	4810

Kappa: 0.9513 (95% CI: 0.94849–0.954034, alpha’s standard error (ASE): 0.001414, *p* < 0.0001). G1: 0.01–0.05, G2: 0.05–0.1, G3: 0.1–0.15, G4: 0.15–0.2, G5: 0.2–0.5, G6: 0.5–1.0, G7: 1.0–1.5, G8: 1.5–2.0, G9: 2.0–2.5, G10: 2.5–4.0, G11: 4.0~. Weighted kappa analysis.

**Table 3:** Estimated PSA value by different assay modalities.

Modality B, PSA Value	Modality A Estimated Value		
	Fitted	95% CI (Lower, Upper)	
0.01	0.021	0.020	0.021
0.02	0.037	0.037	0.038
0.03	0.052	0.052	0.053
0.04	0.067	0.066	0.068
0.05	0.080	0.079	0.081
0.06	0.094	0.093	0.095
0.07	0.107	0.105	0.108
0.08	0.119	0.118	0.121
0.09	0.132	0.130	0.133
0.1	0.144	0.142	0.146
0.11	0.156	0.154	0.158
0.12	0.168	0.166	0.170
0.13	0.180	0.178	0.181
0.14	0.191	0.189	0.193
0.15	0.202	0.200	0.205
0.16	0.214	0.212	0.216
0.17	0.225	0.223	0.227
0.18	0.236	0.234	0.238
0.19	0.247	0.244	0.249
0.2	0.258	0.255	0.260
0.3	0.362	0.359	0.366
0.4	0.462	0.457	0.466
0.5	0.557	0.551	0.562
0.6	0.649	0.642	0.655
0.7	0.738	0.731	0.746
0.8	0.826	0.818	0.834
0.9	0.912	0.903	0.921
1.0	0.996	0.986	1.007
1.1	1.079	1.068	1.091
1.2	1.161	1.149	1.174
1.3	1.242	1.229	1.255
1.4	1.322	1.307	1.336
1.5	1.401	1.385	1.416
1.6	1.479	1.462	1.495
1.7	1.556	1.538	1.574
1.8	1.632	1.614	1.651
1.9	1.708	1.689	1.728
2.0	1.784	1.763	1.804
2.1	1.858	1.836	1.880
2.2	1.932	1.909	1.955
2.3	2.006	1.982	2.030
2.4	2.079	2.054	2.104
2.5	2.151	2.125	2.177
2.6	2.223	2.196	2.251
2.7	2.295	2.267	2.323
2.8	2.366	2.337	2.396
2.9	2.437	2.406	2.467
3.0	2.507	2.476	2.539
3.1	2.577	2.545	2.610
3.2	2.647	2.613	2.681
3.3	2.716	2.681	2.751
3.4	2.785	2.749	2.821
3.5	2.854	2.817	2.891
3.6	2.922	2.884	2.960
3.7	2.990	2.951	3.030
3.8	3.058	3.018	3.098
3.9	3.125	3.084	3.167
4.0	3.192	3.150	3.235
4.5	3.524	3.477	3.573
5.0	3.851	3.797	3.905
5.5	4.171	4.112	4.231
6.0	4.488	4.423	4.553
6.5	4.800	4.730	4.871
7.0	5.108	5.032	5.185
7.5	5.413	5.331	5.496
8.0	5.714	5.627	5.803
8.5	6.013	5.920	6.107
9.0	6.309	6.210	6.409
9.5	6.602	6.497	6.708
10.0	6.892	6.782	7.004

## Data Availability

The datasets used and/or analyzed during the current study are available from the corresponding author on reasonable request.
